# Variation of Seed Yield and Nutritional Quality Traits of Lentil (*Lens culinaris* Medikus) Under Heat and Combined Heat and Drought Stresses

**DOI:** 10.3390/plants14132019

**Published:** 2025-07-01

**Authors:** Hasnae Choukri, Khawla Aloui, Noureddine El Haddad, Kamal Hejjaoui, Abdelaziz Smouni, Shiv Kumar

**Affiliations:** 1 Laboratoire de Biotechnologie et de Physiologie Végétales, Faculté des Sciences, Centre de Recherche BioBio, University Mohammed V, Rabat 10014, Morocco; a.smouni@um5.ac.ma; 2 International Center for Agricultural Research in the Dry Areas (ICARDA), Rabat 10112, Morocco; k.alaoui@cgiar.org; 3 AgroBioSciences Program, College of Agriculture and Environmental Sciences, Mohammed VI Polytechnic, University, Ben Guerir 43150, Morocco; noureddine.elhaddad@um6p.ma (N.E.H.); kamal.hejjaoui@um6p.ma (K.H.); 4 Laboratory of Ecology and Environment, Ben M’Siki Faculty of Sciences, University Hassan II, Casablanca 20800, Morocco; 5 International Center for Agricultural Research in the Dry Areas (ICARDA), Amlaha 466113, India

**Keywords:** seed yield, iron, zinc, protein, cooking time, phytic acid, heat, drought, combined heat and drought, stability

## Abstract

Lentil (*Lens culinaris* Medikus) is a critical food crop offering high protein and essential micronutrients. However, its productivity and nutritional quality are increasingly threatened by climate change. In this study, 36 lentil genotypes were evaluated across two Moroccan locations under normal, heat stress, and combined heat and drought stresses. Significant effects of genotype, environment, and their interactions were observed on seed yield, seed size, cooking time, and nutritional quality. Heat and drought stresses caused substantial reductions in seed yield (up to 40% under combined stress), protein content, iron, and zinc concentration, and increased phytic acid levels, which negatively impacted iron and zinc bioavailability. Cooking time significantly decreased under stress conditions, with up to 54% reduction under combined heat and drought stresses at Annoceur research station. Correlation analysis revealed complex trade-offs among yield, nutritional quality, and cooking traits under stress conditions. Principal component analysis and GGE biplot analyses identified genotypes with superior yield, micronutrient concentration, and cooking time stability across environments. Genotypes such as G32, G3, and G36 combined high iron and zinc levels; G13 and G30 showed low phytic acid, while G 15 exhibited the shortest cooking time. These genotypes also demonstrated adaptability across the tested environment. This study highlights the potential of selecting climate-resilient, nutrient-dense lentil genotypes to support breeding efforts aimed at improving food security in the face of global climate variability. These genotypes can be suggested as elite climate-resilient parental lines to support breeders in enhancing lentil yield, nutritional quality, and stability under multiple stress conditions.

## 1. Introduction

Lentil (*Lens culinaris* Medikus) is an important staple food crop. It is a rich source of protein (20–36%) and contains essential micronutrients and vitamins in a highly bioavailable form to the human body. In addition to its nutritional value, lentils generally have a fast cooking time compared to other legumes, due to its smaller seed size and thin seed coat [1]. Cooking time duration holds a significant importance in breeding programs, and a prolonged cooking time for a new variety could lead to market penalties due to lower demand. Conversely, a fast-cooking variety may achieve higher prices due to the convenience it offers in cooking and reduced energy costs [2]. Lentil is grown over 5.5 million hectares, with over 6.6 million tonnes of production worldwide [3]. Lentil is a resilient crop capable of growing in diverse climatic and soil conditions. It is predominantly cultivated in mediterranean and subtropical dryland regions, where its ability to fix atmospheric nitrogen (N_2_) minimizes the reliance on synthetic fertilizers. However, global lentil productivity has declined in recent decades due to climate fluctuations, including extreme temperatures, flooding, drought, and an increased incidence of pests and diseases [4]. Multiple stresses such as heat and drought during the seed filling stage adversely affect lentil productivity. Considerable variation was observed in lentil response to abiotic stresses across different agro-ecological regions. In South Asia, lentil is grown on residual moisture soil as a post-rainy season crop. As a result, early withdrawal of rains during crop establishment significantly impacts germination and early growth. Conversely, growing lentil in West Asia is often subject to terminal drought and heat stress during the reproductive phase, involving different adaptive traits [5]. Similarly, in Sub-Saharan Africa, lentil is cultivated under rainfed conditions and often experience significant yield reductions due to water stress during critical growth stages [6]. This regional variability has been well documented and highlights the importance of multi-environment testing to identify broadly adapted, stress-resilient genotypes suited for a changing climate [7]. On the other hand, increased heat intensity and water deficits have a substantial impact on lentil nutritional quality. Several studies have reported that iron and zinc concentration in lentil seed was reduced due to heat and drought conditions [5,8]. Protein concentration in the seeds was also affected. To attain global food security under changing climate, lentil breeders face a significant challenge of simultaneously enhancing both yield and nutritional quality.

Several studies reported a negative correlation between seed yield and different quality traits including iron, zinc and protein concentration in lentil [7,9]. The genetic and environmental factors result in differences in both agronomic performance and seed nutritional properties in lentils [10,11,12]. Several research underlined that genotype and growing environment largely affect micronutrient, protein concentration, and cooking time of lentil varieties [13]. In addition, stability analysis is crucial in plant breeding to assess the performance of new or/and enhanced genotypes across a range of test environments. One effective tool used is the “Which-Win-Where plot, which visually represents genotype performance across different environments. This plot helps identify the best-performing genotypes in specific conditions, highlighting both their adaptability and stability. However, the stability of yield and nutritional quality traits under climate change is complex and requires further investigation.

This study aimed to (i) investigate the environmental and genotypic effect on seed yield, crude protein (CP), micronutrients (iron and zinc), phytic acid, and cooking time of 36 lentil genotypes grown at two diverse locations under three treatments (normal, heat, and drought stresses), (ii) assess the stability of seed yield and key nutritional quality traits of lentil, and (iii) identify superior genotypes with higher seed yield and nutritional values, which could be further suggested as parental material in breeding programs.

## 2. Results

### 2.1. Analysis of Variance

The ANOVA Table 1 and Table 2 presented the effects of genotype (G), treatment (T), and location (L) on seed yield, seed size, and seed nutritional quality (Fe, Zn, PA/Fe, PA/Zn, PA, PC, and CT) of 36 genotypes that were grown in two locations, Anneucer and Merchouch, under three environmental stress conditions: normal, heat, and heat combined with drought. The analysis was conducted separately for each location and then combined. Genotype had a significant effect (*p* < 0.01) on most measured traits across both locations. Treatment had a highly significant effect, with the lowest *p*-values observed for iron concentration, PA/Fe molar ratio, and protein concentration. In the combined analysis, location significantly affected all traits. The interaction between genotype and treatment (G × T) was significant for most traits. The genotype × location (G × L) interaction was significant for several traits. The combined analysis showed significant genotype × treatment × location interactions for several traits.

### 2.2. Mean Performance for Seed Yield, Seed Size and Shape Parameters

According to the mean comparison presented in Table 3, seed yield differed significantly across the tested environments (*p* < 0.001). Under E1, seed yield ranged from 2.97 to 6.93 g, with a mean of 5.13 g. In E2, where heat stress was applied, seed yield decreased by 24%. In E3, combining heat stress and drought, seed yield was drastically reduced by 46%. In E4 (normal planting conditions), the mean seed yield was 4.04 g, lower than E1 but still stable. Heat stress under E5 caused a 20% reduction, with yields ranging from 1.76 to 4.73 g, while in E6, the yield dropped by 38%.

For hundred seed weight, E1 recorded the highest mean of 3.50 g, with a range from 0.69 to 4.90 g. E2 showed a significant reduction of 27%, with a mean of 2.55 g. The most substantial decrease occurred in E3, where the hundred seed weight dropped by 51% to a mean of 1.73 g. In E4, the hundred seed weight was 2.46 g, still higher than later environments. The hundred seed weight continued to decline in E5 and E6, with reductions of 22% and 35%, resulting in mean values of 1.93 g and 1.60 g, respectively.

Seed length remained relatively stable across environments. In E1, the mean was 4.70 mm, with a range from 0.61 to 5.83 mm. E2 showed a slight reduction of 2%, bringing the mean to 4.60 mm. E3 had a minimal reduction of 1%, with a mean of 4.65 mm. E4 showed the highest mean value of 4.78 mm, while E5 and E6 saw a small increase, with means of 4.83 mm and 4.81 mm, respectively, indicating that seed length was less impacted by environmental stresses.

Seed width displayed minor fluctuations across environments. In E1, the mean was 4.43 mm, with a range from 0.61 to 5.41 mm. E2 and E3 showed small reductions of 3% and 2%, with means of 4.29 mm and 4.35 mm, respectively. E4 had a slightly lower mean of 4.25 mm; however, in E5 and E6, seed width increased slightly to 4.48 mm and 4.47 mm, showing a 5% increase compared to E4, though still 5% lower than in E1.

Seed area showed consistency across environments, though slight reductions were observed. In E1, the mean was 16.16 mm^2^, with a range from 4.36 to 24.39 mm^2^. E2 and E3 showed minimal reductions of 2%, with means of 15.81 mm^2^ and 15.86 mm^2^, respectively. In E4, seed area increased to 17.45 mm^2^, representing the highest mean across all environments. E5 and E6 showed slight decreases of 4% and 5%, with means of 16.70 mm^2^ and 16.62 mm^2^, respectively.

Seed diameter showed high stability across environments. In E1, the mean was 4.48 mm, with a range from 0.61 to 5.47 mm. In E2 and E3, SD decreased slightly by 2%, with means of 4.39 mm and 4.37 mm, respectively. E4 had the highest mean of 4.65 mm, while in E5 and E6, seed diameter slightly decreased to 4.57 mm and 4.56 mm, reflecting minimal variation.

Seed perimeter followed a similar trend to SD, with minimal changes across environments. In E1, the mean was 17.59 mm. E2 showed a slight decrease of 1% (mean: 17.39 mm), while E3 had a marginal reduction of less than 1%, with a mean of 17.46 mm. The highest seed perimeter was recorded in E4, with a mean of 19.27 mm, and a noticeable decrease of about 7% was observed in E5 and E6, with means of 17.99 mm and 17.95 mm, respectively.

### 2.3. Mean Performance of Nutritional Quality and Micronutrient Bioavailability

The range and mean values of nutritional traits in each environment are provided in Table S2, iron concentration varied significantly across environments, in E1 the range was from 7.27 to 11.08 g/100 g, with a mean of 8.72 g/100 g. Compared to the other environments, iron concentration showed a wide range of variation in E4, ranging from 7.14 to 11.86 g/100 g, with a the highest mean of 10.22 g/100 g. Heat stress conditions in both E2 and E5 resulted in a reduction in iron concentration (24% and 18%, respectively). The mean iron concentration was 7.61 g/100 g in E2, while in E5 the mean was 8.46 g/100 g. On the other hand, E3 exhibited a lower iron concentration compared to both E1 and E2. Iron concentration ranged from 5.33 to 8.03 g/100 g, with a mean of 6.57 g/100 g. Heat stress combined with drought conditions in this environment (E3) led to a reduction of 25% in iron content, while it showed a substantial reduction of 34% in E6.

Zinc concentration in E1 ranged from 5.04 to 6.79 g/100 g, with a mean of 5.89 g/100 g. Comparatively, E4 displayed a wide range of zinc concentration, varying from 4.83 to 6.71 g/100 g with a mean of 5.66 g/100 g. The incidence of heat stress conditions in both E2 and E5 resulted in reductions in zinc concentration, with decreases of 4% and 3%, respectively. E2 had a mean zinc concentration of 5.66 g/100 g, while E5 had a mean of 5.11 g/100 g. In contrast, E3 exhibited lower zinc concentration compared to E1 and E2, ranging from 4.47 to 6.61 g/100 g, with a mean of 5.24 g/100 g. In this environment, the combination of heat stress and drought conditions led to a reduction of 28% in zinc concentration. Similarly, E6 experienced a reduction of 7% in iron concentration.

Cooking time was significantly reduced (28%) under the E2 condition, the mean was 7.05 min, varying from 4.82 to 9.98 min. Similarly, in E3, cooking time showed a decrease of 48%. The mean cooking time was 5.04 min only. Cooking time in E1 showed the longest duration, ranging from 6.17 to 14.94 min, with a mean of 9.82 min. In E4, the range of cooking time was between 5.30 and 14.21 min, with a mean of 9.68 min. Similarly to E1, E4 has a relatively wide range of cooking time. On the other hand, cooking time showed a reduction of 37% in E5, and the range was from 4.15 to 8.71 min, with a mean of 6.05 min. Similarly, cooking time was further decreased (54%) under E6. The range of cooking time was from 2.54 to 6.21 min, with a mean of 4.43 min in E6.

The protein concentration exhibited a notable variation across the six tested environments (E1 to E6). Environment E1 demonstrated the highest average protein content, with a mean of 29.66 g/100 g and a range from 28.75 to 31.98 g/100 g, closely followed by E4, which had a mean of 28.96 g/100 g and a range of 27.13 to 31.24 g/100 g. These environments provided the most favorable conditions for protein synthesis and accumulation in lentil seeds. In contrast, E6 showed the lowest protein content, with a mean of 21.74 g/100 g and a narrower range of 19.61 to 23.71 g/100 g. E2 and E3 exhibited moderate protein levels with means of 27.41 g/100 g and 25.67 g/100 g, respectively, and slightly overlapping ranges. E5 presented a lower mean protein concentration of 24.54 g/100 g, within a broader range of 22.12 to 28.31 g/100 g.

The range of phytic acid concentration in E1 was 0.54 to 0.98 g/100 g, with a mean of 0.82 g/100 g. This environment exhibited relatively low phytic acid levels. The E2 environment has increased phytic acid concentration compared to E1, with a range from 0.82 to 1.20 g/100 g and a mean of 0.99 g/100 g. Similarly, E3 demonstrated a further increase in phytic acid level, and the range was 0.98 to 1.39 g/100 g with a mean of 1.20 g/100 g.

**Figure 1:**
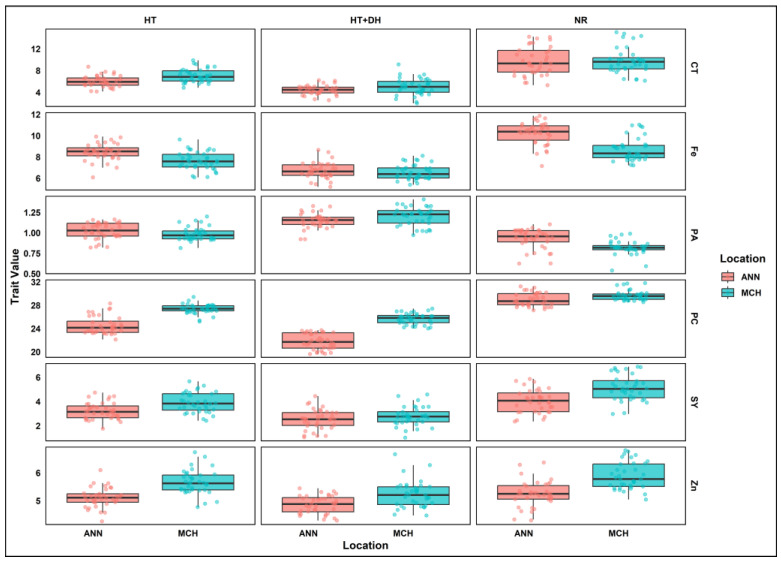
Grouped boxplots of lentil seed yield and nutritional quality traits variation across treatments and locations. SY, Seed yield; CT, Cooking time; PC, Protein concentration; PA, Phytic acid; Zn, Zinc; Fe, Iron; MCH, Marchouch; ANN, Annoceur; NR, Normal condition; HT, Heat stress; HT+DH, Combined heat and drought stresses.

A similar pattern was also observed across E4, E5, and E6. In E4, the range of phytic acid concentration was between 0.62 and 1.10 g/100 g, with a mean of 0.93 g/100 g. Lentil seeds from this environment have a relatively lower range of phytic acid concentration. Similarly, E3 and E5 showed an increase of 14% in phytic acid levels, and the range was between 0.82 and 1.44 g/100 g, with a mean of 1.10 g/100 g. In E6, the phytic acid levels range from 0.92 and 1.33 g/100 g, with a mean of 1.15 g/100 g (Figure 1).

In E1 and E4, phytic acid/micronutrients molar ratios of both iron and zinc showed reduced value compared to heat stress conditions in E3 and E5, where PA/Fe and PA/Zn molar ratios showed an increase of 38% and 25%, respectively. Under E3, both molar ratios showed a significant increase of 95% for PA/Fe and 66% for PA/Zn. Similarly, E6 conditions resulted in an increase of 89% in PA/Fe molar ratio, while PA/Zn ratio showed an increase of 34%.

### 2.4. Correlation Between Seed Yield, Seed Size/Shape, and Nutritional Quality Traits

**Figure 2:**
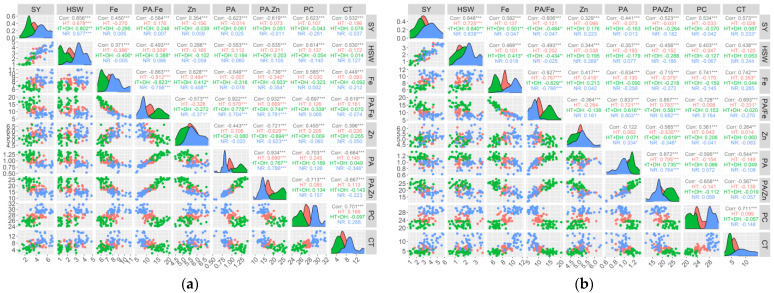
Pearson correlations between seed yield and seed nutritional traits under three heat, drought and combined heat and drought across Marchouch (**a**) and Annoceur (**b**) research stations. SY, Seed yield; HSW, hundred seed weight; CT, Cooking time; PC, Protein concentration; PA, Phytic acid; Zn, Zinc; PA/Zn, Phytic acid/Zinc ratio; Fe, Iron; PA/Fe, Phytic acid/Iron ratio; Normal condition; HT, Heat stress; HT+DH, Combined heat and drought stresses;*, **, and *** indicate significance at 0.05, 0.01, and 0.001 probability levels, respectively.

Significant positive and negative correlations were detected between studied traits (Table S3). Under E1 conditions, seed yield showed positive and highly significant correlation with hundred seed weight (r = 0.69; *p* < 0.001), and significant negative correlation with protein concentration (r = −0.29; *p* < 0.5). Iron concentration revealed a significant negative correlation with zinc (r = −0.48; *p* < 0.001), whereas there was a significant negative correlation between iron concentration and both PA/Fe (r = −0.75; *p* < 0.001) and PA/Zn (r = −0.36; *p* < 0.1) ratios. PA/Fe ratio showed a positive and highly significant correlation with both phytic acid (r = 0.71; *p* < 0.001) and PA/Zn ratio (r = 0.79; *p* < 0.001), while PA/Fe was negatively correlated with zinc concentration (r = −0.38; *p* < 0.001). Zinc concentration was also negatively correlated with PA/Zn ratio (r = −0.62; *p* < 0.001). Similarly, PA/Zn was negatively correlated with phytic acid (r = 0.80; *p* < 0.001). In contrast, phytic acid revealed a significant negative correlation with cooking time (r = −0.34; *p* < 0.01). Another positive and highly significant correlation was observed between cooking time and protein concentration (r = 0.33; *p* < 0.01). Under E2 conditions, seed yield showed a positive and significant correlation with the hundred seed weight (r = 0.68; *p* < 0.001) and significant negative correlation with iron concentration (r = −0.28; *p* < 0.05). Similarly, hundred seed yield was also negatively correlated with iron concentration (r = −0.38; *p* < 0.001). A significant positive association was obtained between the hundred seed weight and the PA/Fe ratio (r = 0.37; *p* < 0.01). With respect to iron content, a negative and significant correlation was obtained with PA/Fe (r = −0.83; *p* < 0.001) and PA/Zn (r = −0.36; *p* < 0.01). On the other hand, iron concentration revealed a highly positive and significant correlation with zinc concentration (r = 0.48; *p* < 0.001). PA/Fe ratio showed a highly positive and significant correlation with phytic acid (r = 0.58; *p* < 0.001) and PA/Zn ratio (r = 0.68; *p* < 0.001), while there was a negative correlation zinc concentration (r = −0.31; *p* < 0.01). As for the PA/Zn ratio, a highly significant positive correlation was observed with phytic acid (r = 0.70; *p* < 0.001), while the association with zinc was negative and highly significant (r = −0.60; *p* < 0.001). Interestingly, phytic acid showed a positive correlation with protein concentration (r = 0.24; *p* < 0.5), while the correlation with cooking time was positive but not significant. Coming to E3, there was a significant positive correlation between seed yield and hundred seed weight (r = 0.80; *p* < 0.001) as well as with PA/Fe (r = 0.24; *p* < 0.05), whereas seed yield was negatively correlated with iron concentration (r = −0.29; *p* < 0.01). Hundred seed weight revealed a negative significant correlation with iron concentration (r = −0.40; *p* < 0.001) and positive correlation with PA/Fe ratio (r = 0.38; *p* < 0.01). A significant negative association was identified between iron and PA/Fe (r = −0.83; *p* < 0.001), phytic acid (r = −0.32; *p* < 0.01), PA/Zn (r = −0.51; *p* < 0.001), and protein concentration (r = −0.26; *p* < 0.05). Zinc concentration revealed a positive correlation between iron concentration (r = 0.42; *p* < 0.001) and cooking time (r = 0.26; *p* < 0.05), while a highly significant negative correlation was observed with PA/Zn ratio (r = −0.70; *p* < 0.001). The correlation between phytic acid and PA/Zn was positive and highly significant (r = −0.75; *p* < 0.001); however, a non-significant correlation was observed between phytic acid and cooking time. For E4, seed yield was positively correlated with the hundred seed weight (r = 0.80; *p* < 0.001) and cooking time (r = 0.31; *p* < 0.01). Iron concentration showed a positive correlation with cooking time (r = 0.29; *p* < 0.05), while there was no significant correlation with zinc content. In contrast, zinc concentration was positively correlated with phytic acid (r = 0.25; *p* < 0.05) When looking at phytic acid/micronutrient molar ratio, PA/Fe showed a highly significant positive correlation with phytic acid (r = 0.80; *p* < 0.001) and PA/Zn ratio (r = 0.63; *p* < 0.001), whereas this ratio showed a significant negative correlation with iron (r = −0.75; *p* < 0.001) and cooking time (r = −0.25; *p* < 0.05). The PA/Zn ratio displayed a highly significant positive correlation with phytic acid (r = 0.72; *p* < 0.001) and a highly significant negative correlation with zinc concentration (r = −0.49; *p* < 0.001). For E5, seed yield was highly and positively correlated with the hundred seed yield only (r = 0.70; *p* < 0.001). Another positive correlation was observed between iron concentration and both zinc concentration (r = 0.33; *p* < 0.01) and cooking time (r = 0.29; *p* < 0.05). On the other hand, PA/Fe displayed a significant negative correlation with iron concentration (r = −0.75; *p* < 0.01) and cooking time (r = −0.31; *p* < 0.01), while there was a highly significant positive correlation with phytic acid (r = 0.73; *p* < 0.001) and PA/Zn ratio (r = 0.73; *p* < 0.001). Similarly, PA/Zn ratio also showed a highly significant positive correlation with phytic acid (r = 0.73; *p* < 0.01), whereas there was a significant negative correlation with iron (r = −0.34; *p* < 0.01) and zinc concentration (r = −0.57; *p* < 0.001). In E6, seed yield was positively correlated with hundred seed yield (r = 0.76; p < 0.001) and iron concentration (r = −0.44; *p* < 0.001). The hundred seed yield also showed a positive correlation with iron concentration (r = 0.33; *p* < 0.01). As for phytic acid/micronutrients, PA/Fe revealed a highly significant positive correlation with phytic acid (r = 0.66; *p* < 0.001) and PA/Zn ratio (r = 0.59; *p* < 0.001), while this ratio showed a highly significant negative association with iron content. Similarly, PA/Zn ratio demonstrated a highly significant positive correlation with phytic acid (r = 0.70; *p* < 0.001). Under this environment, cooking time displayed a positive but non-significant correlation with phytic acid (Figure 2).

### 2.5. Principal Component Analysis (PCA) of Lentil Genotypes Across Environments

**Figure 3:**
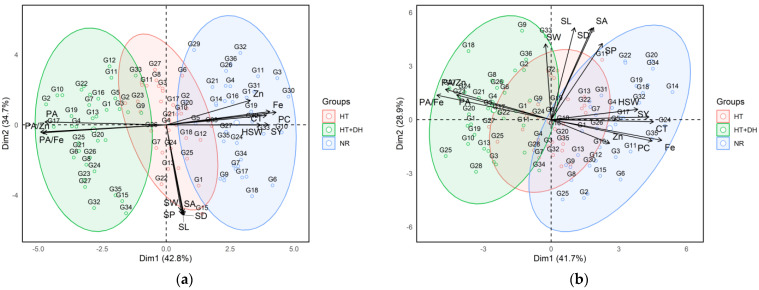
Principal component analysis and traits contributing to the variability under normal, heat, and combined heat and drought stress conditions across Marchouch (**a**) and Annoceur (**b**) research stations. Fe, Iron; Zn, Zinc; PA, Phytic acid; PC, protein concentration; CT, Cooking time; SY, Seed yield; HSW, hundred seed weight; SL, Seed length; SW, Seed width; SA, Seed area; SP, Seed perimeter; SD, Seed diameter, HT; heat stress, HT + DH, combined heat and drought; NR, normal condition.

The two Principal Component Analysis (PCA) biplots illustrated the multivariate relationships among lentil genotypes based on their agronomic, morphological, and nutritional traits under different environmental stress conditions at two experimental stations: Marchouch and Annoceur (Figure 3). The first biplot corresponded to the Marchouch station, where the first two principal components (PC1 and PC2) account for 77.5% of the total variance (42.8% and 34.7%, respectively). In this plot, genotypes were clearly grouped based on their exposure to different environments: normal (NR), heat stress (HT), and combined heat and drought stresses (HT + DH). Genotypes cultivated under normal conditions (blue cluster) are mostly located on the left side of the biplot and showed strong positive associations with yield-related traits, such as seed yield, hundred seed weight and nutritional quality parameters like iron and zinc concentration, protein concentration, and cooking time. In contrast, genotypes exposed to combined heat and drought stresses (green cluster) are positioned on the right side and are more closely associated with antinutritional traits, particularly phytic acid and its molar ratios with iron and zinc (PA/Fe and PA/Zn). The heat stress group (red cluster), situated between the normal and combined heat stress and drought stresses clusters, tended to associate more with morphological traits like seed area, seed width, seed length, seed perimeter, and seed diameter.

The second PCA biplot, derived from the Annoceur station, showed a similar structure with the first two principal components explaining 70.6% of the total variance (41.7% for PC1 and 28.9% for PC2). Despite some overlap, the clustering pattern of genotypes remained consistent, reinforcing the influence of environmental stress on trait expression. As in Marchouch, under normal conditions, genotypes (blue) in Annoceur exhibited positive correlations with seed yield, hundred seed weight, iron, zinc, protein concentration, and cooking time. Meanwhile, genotypes grown under combined heat stress and drought stresses (green) again aligned with phytic acid and its associated molar ratios. The heat stress group (red) at Annoceur was also clustered near seed morphological traits, reflecting the trend observed in Marchouch. Importantly, the trait vectors in both biplots showed consistent orientation, nutritional traits, and antinutritional factors are generally opposed, highlighting a trade-off between nutrient concentration and bioavailability under stress.

### 2.6. GGE Biplots Based Analysis

#### 2.6.1. Mean Vs. Stability

**Figure 4:**
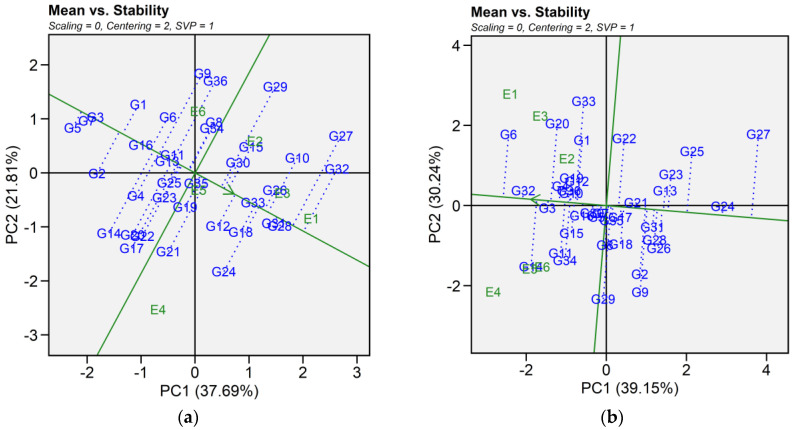

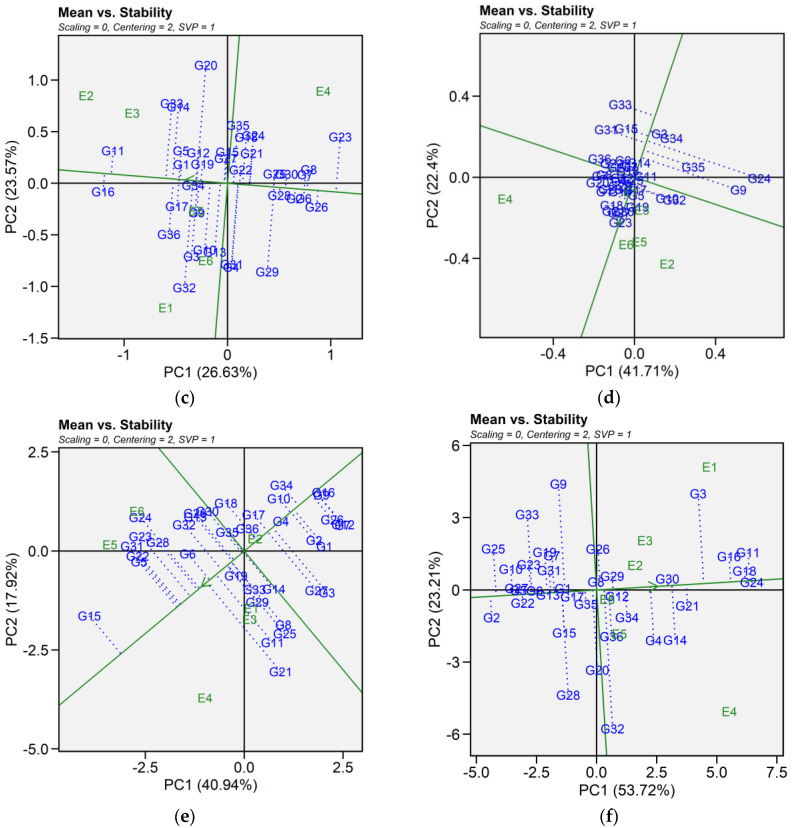
Patterns (**a**–**f**). Mean vs. stability based on PC1 and PC2 illustrating G × E interactions of the 36 lentil accessions under six environments. (**a**) SY, Seed yield; (**b**) Fe, Iron; (**c**) Zn, Zinc; (**d**) PA, Phytic acid; (**e**) PC, Protein concentration; (**f**) CT, Cooking time. The mean vs. stability was based on scaling = 0, centering = 2, and singular value partitioning (SVP) = 1.

The mean vs. stability pattern explained 59.50% for seed yield, 69.39% for iron, 50.20% for zinc, 64.11% for phytic acid, 58.86% for crude protein, and 76.93% for cooking time (Figure 4). For seed yield, G34, G36, G27, G10, G4, G16, and G2 performed well in E1 and E2, while G9, G36, and G29 yielded the most in E3 and E6. G17, G24, G21, G14, G20, G22, and G12 excelled in E4 and E5, while G5, G7, and G3 were most stable despite lower yields. High-yielding genotypes such as G24, G27, and G32 exhibited instability. For iron, G14, G34, G11, G29, G32, G3, G15, and G6 had the highest concentrations in E4 and performed well in E5 and E6. In E1, E2, and E3, G6, G20, G33, G1, G32, G19, G22, G3, G4, and G12 excelled. Stable genotypes included G3, G32, G36, G10, and G4, whereas G29, G9, G14, and G34 were less stable. For zinc, G32, G10, G36, G3, G4, and G13 had high levels in E1, E5, and E6. In E2 and E3, G16, G11, G14, G33, G5, G9, G34, and G12 performed best, while in E4, G23, G8, G7, G6, G26, G30, G2, and G25 had higher levels. G34 and G19 were the most stable, while G25 was stable but had lower zinc content. For phytic acid, G13, G34, G33, G2, G1, G10, G36, G30, G22, and G21 had lower levels in E1, E5, and E6, with G30, G21, G2, and G13 being the most stable. In E2 and E3, G13, G1, G2, G9, G32, G11, G36, and G22 had lower levels, while in E4, G34, G10, G15, G5, G33, G7, G8, and G16 performed best. G13 and G5 showed the highest stability across E2, E3, and E4. For protein content, G21, G11, G25, G8, G29, G14, G33, and G15 had higher levels in E1, E2, and E4. In E6 and E5, G15, G24, G31, G23, G22, G5, G28, G32, and G13 showed higher levels, while E3 saw moderately higher levels in G7, G12, G26, G1, G16, G3, G9, and G2. G3 was the most stable genotype with high protein content. For cooking time, G25, G33, G9, G10, G23, G19, G27, and G2 cooked faster in E4, E5, and E6. In E1, E2, and E4, G28, G32, G2, G22, G27, and G5 had shorter cooking times. Across environments, G29, G8, G12, and G30 were more stable but exhibited longer cooking time (Table S4).

#### 2.6.2. Ranking Genotypes

**Figure 5:**
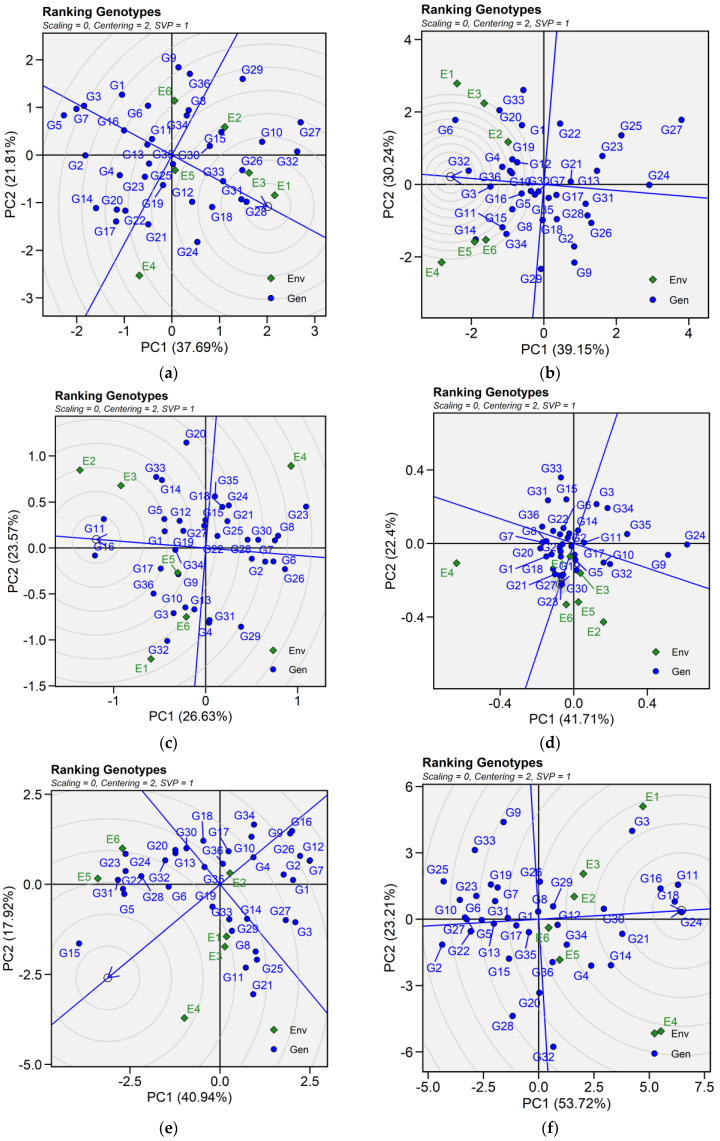
Patterns (**a**–**f**). Ranking genotypes biplots based on PC1 and PC2 illustrating G × E interactions of the 36 lentil accessions under six environments. (**a**) SY, Seed yield; (**b**) Fe, Iron; (**c**) Zn, Zinc; (**d**) PA, Phytic acid; (**e**) PC, Protein concentration; (**f**) CT, Cooking time. The mean vs. stability was based on scaling = 0, centering = 2, and singular value partitioning (SVP) = 1.

The biplot facilitated the identification of superior and ideal genotype from the pool of 36 tested genotypes. An ideal genotype is localized within the innermost circle, positioned close to the arrowhead at the center of the circular ring. However, in case no genotype was located inside the inner circle, genotypes next to the inner circle are ideal ones. For seed yield, genotypes G28, G31, G26, and G33 were regarded as the best genotypes. Similarly, genotypes G32, G3, G4, and G36 are ideal for iron content. For zinc content, G17, G3, G36, G11, G32 and G9; for phytic acid, G23, G21, G27. Genotypes G24, G18, G16, and G11 were identified best genotypes for protein concentration across tested environments. As for cooking time, only genotype G15 was suitable for evaluated environments (Figure 5).

#### 2.6.3. Which-Won-Where and Mega-Environment Identification

**Figure 6:**
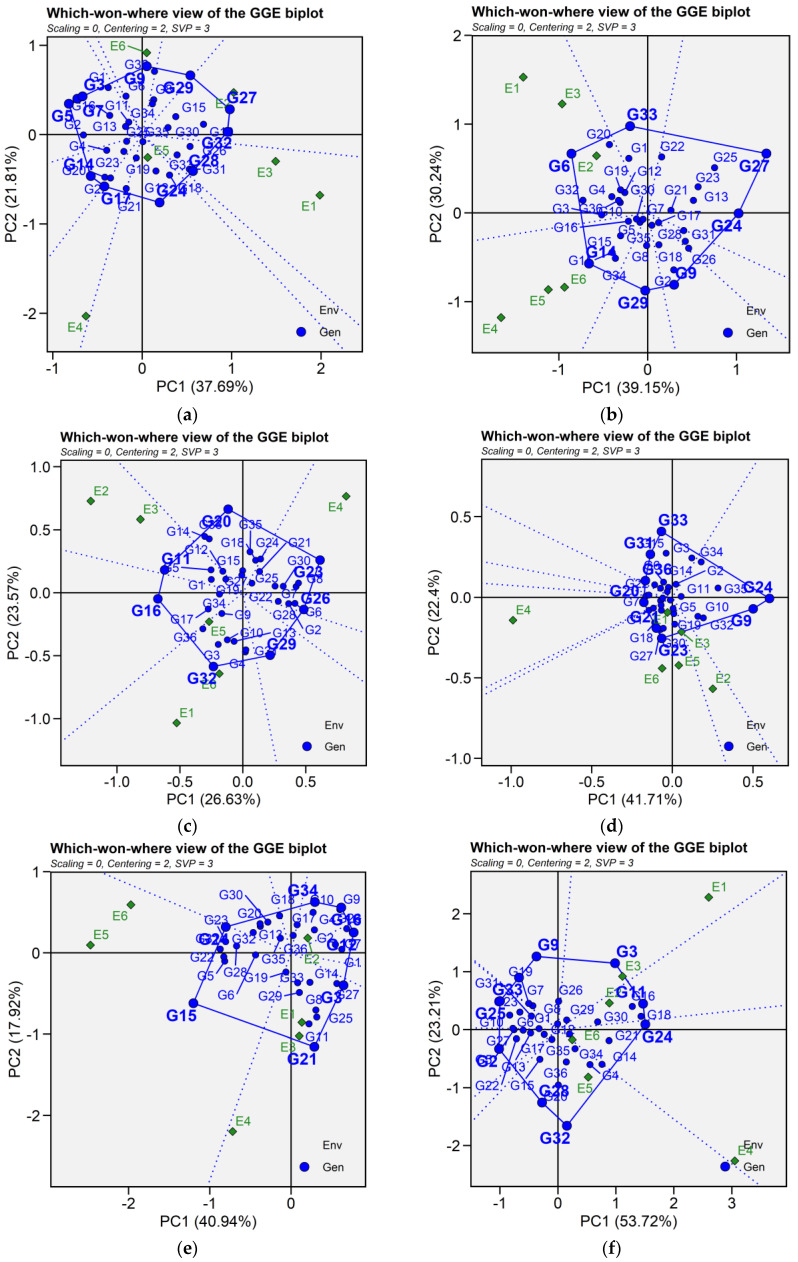
Patterns (**a**–**f**). Which-won-where view of GGE biplots based on PC1 and PC2 illustrating G × E interactions of the 36 lentil accessions under six environments. (**a**) SY, Seed yield; (**b**) Fe, Iron; (**c**) Zn, Zinc; (**d**) PA, Phytic acid; (**e**) PC, Protein concentration; (**f**) CT, Cooking time. The mean vs. stability was based on scaling = 0, centering = 2, and singular value partitioning (SVP) = 1.

Crossover GEI, mega-environment differentiation and specific adaptation of genotypes are graphically represented by GGE biplot for which-won-where (Figure 6). In this graph, a polygon is drawn joining the genotypes that are located distant from the biplot origin so that all other genotypes are contained in the polygon. The vertex genotypes are characterized by the longest vectors in their respective directions, serving as an indicator of their responsiveness to environments. For seed yield, the polygon is formed by connecting the following vertex genotypes G5, G7, G3, G9, G29, G27, G32, G24, G17, and G14. For iron concentration, the polygon is formed by genotype G14, G29, G9, G24, G27, G33, and G6. For zinc content, genotype G32, G10, G6, G23, G20, G14, and G16 are joining together to form the polygon. Similarly, for phytic acid, the vertex genotypes were identified as genotypes G33, G34, G35, G24, G32, G23, G1, G7, G36, and G31. For protein content, genotypes G9, G3, G11, G24, G32, G28, G25, and G33 were connected to form the polygon. As for cooking time, the vertex genotypes G21, G15, G24, G34, G16, G12, G7, G3 and G21 formed the polygon. The equality lines to the sides of the polygon divide the biplot into sectors, and thereby the biplot subdivides the target environment into subregions (mega-environments). Mega-environments are those sectors that include one or more environments. The perpendicular lines divided the biplot into eight sectors for seed yield, iron concentration, and cooking time; six sectors for zinc content; ten sectors for phytic acid; and seven sectors for protein content. For seed yield, test environments were grouped into three mega-environments, where E1, E2, and E3 as well as genotypes G27, G29, and G32 fell into the first mega-environment; these genotypes were the most adapted and highest yielding across the three environments. Similarly, E4 and E5 fell into the second mega-environment with G17 and G24 the most adapted, while E6 fell along with G9 into one mega-environment. For iron content, the first mega-environments included environment E1, E2, E3 as well as G6 and G33, while the second one included environments E4, E5 and E6 along with G14 as the nominal winner. Three mega-environments were identified for zinc content, with environment E3, E2, and E5 grouped together in a mega-environment with G16 as the best performing genotype. Environment E1 and E6 were in the second mega-environment along with G32 and G10 ranking higher in zinc content, while the third mega-environment included E4 and G23. Similarly, there were three mega-environments for phytic acid, the first one contained environment E5, E6, and E1 and G23 as winner genotype. Environments E2, E3, G32 and G24 constituted the second mega-environment, whereas E4 and G1 fell into the third mega-environment. As for protein content, the first mega-environment comprised E4, E5, and E6 along with genotypes G24 and G15, the second mega-environment contained E1, E2, and genotypes G21, while environment E3 fell into the third mega-environment beside G12. Regarding cooking time, two mega-environments were identified; E4, E5, E6 and G32 were presented in one mega-environment, and the second mega-environment involved E1, E2, and E3 as well as genotypes G11, G3, and G24 (Figure 6).

## 3. Discussion

Abiotic stresses, such as heat and heat combined with drought, are the major challenges to agriculture in various production regions. Heat and combined heat and drought stress conditions, accruing during reproductive stage of lentil, revealed significant negative impact on morphological and agronomical traits, which negatively impact grain yield and nutritional quality. Developing high yielding nutrient dense cultivars combined with high stability across varying heat and heat combined with drought levels is the primary goal of modern breeding programs. Our previous studies reported that heat and heat combined with drought conditions induced a significant reduction in seed yield, seed size, nutritional quality traits [5,7]. Moreover, heat stress has been linked to a significant accumulation of phytic acid in lentil seeds [8]. However, promising genotypes have been identified, under controlled conditions, and were characterized by high yielding short cooking time, high iron, and moderate zinc bioavailability [8]. In this study, 36 lentil genotypes were tested at two different locations, The late planting approach was implemented to ensure exposure of the flowering and pod-filling stages to heat stress (E2 and E5), to sustain heat stress conditions, the high-temperature treatment was supplemented with frequent irrigation (E3 and E6).Varied weather conditions were observed in tested environments, highest precipitation was recorded during January (42 mm) in Marchouch station, whereas higher temperatures (36 °C) were recorded during July in Annoceur station. The observed precipitation and temperature levels varied across environments and eventually had a significant effect on the seed yield and nutritional quality parameters of tested genotypes.

This study demonstrated significant genetic variation among the evaluated genotypes for micronutrients (iron and zinc), protein content, cooking time, and antinutrient (phytic acid) under both normal and stress conditions, indicating a promising potential for selecting high levels of these quality traits. Similar results also reported the significant variation of iron and zinc of genotypes tested under different locations [14,15].Grain yield variation under normal and stress conditions across the tested environments highlighted the potential for selecting high-yielding genotypes with enhanced Fe, Zn, and protein concentration in the seeds, in addition to low phytic acid level and short cooking.

The nutritional traits of lentil genotypes varied significantly across environments. Our fundings were in agreement with the result reported by Chengci et al. (2022) [16].Iron concentration ranged from 5.33 to 11.86 g/100 g, with the highest mean (10.22 g/100 g) in E4 and the lowest (6.57 g/100 g) in E3. Heat and drought stresses reduced iron concentration by up to 34% in E6. Zinc concentration followed a similar trend, with E1 showing the highest mean (5.89 g/100 g) and E3 experiencing a 28% reduction. Cooking time varied significantly, with the longest duration in E1 (9.82 min) and the shortest in E6 (4.43 min), indicating a 54% reduction under extreme heat and drought. Protein concentration was highest in E1 (29.66%) and lowest in E6 (21.74%), reflecting the impact of environmental stress on protein accumulation. Phytic acid (PA) levels increased under heat stress, particularly in E3 and E5, where PA/Fe and PA/Zn molar ratios rose by 95% and 66%, respectively. Our previous findings have also highlighted the impact of heat and drought stresses on iron, zinc, protein content, cooking time, and phytic acid [5,6,7].

Correlation analysis in our study revealed that seed yield was highly correlated with hundred seed yield across all tested environments. The relationship between iron concentration and seed yield varied across environments, indicating the influence of environmental stress on nutrient accumulation. Iron concentration showed non-significant correlation with seed yield under E1 (No stress) conditions. Similarly, the association between iron concentration and seed yield remained non-significant under both E4 (No stress) and E5 (Heat stress) environmental conditions which is consistent with our previous findings [5,7]. Conversely, seed yield showed a significant negative correlation with iron concentration in E2 (Heat stress) and E3 (Heat-drought stress), while a significant positive correlation was observed under E6 (Heat-drought stress) growing conditions. These findings highlight the complexity of nutrient accumulation under stress conditions and suggest that selecting high-yielding genotypes with stable micronutrient concentration remains a challenge in breeding programs. Across tested environmental conditions, seed yield showed a non-significant correlation with zinc concentration, which is in agreement with our findings. This suggests that zinc accumulation is relatively independent of yield performance, making it necessary to incorporate targeted selection strategies for zinc biofortification [17]. In the same way, many researchers reported similar observation in lentil. Similarly to our previous outcomes, phytic acid also demonstrated a non-significant correlation with grain yield under all environmental conditions, reinforcing the idea that reducing phytic acid levels to improve iron and zinc bioavailability does not necessarily impact yield performance. A positive significant correlation was noticed between seed yield and protein concentration under environment E1, while under other environment the correlation was non-significant, suggesting that stress conditions may disrupt the positive association typically observed under optimal growing conditions. Seed yield also revealed a positive correlation with cooking time under environment E4, however there was a non-signification correlation under other environments. A positive correlation was observed between seed iron and zinc concentrations under environment E1, E2, and E3, suggesting that selection for one micronutrient may lead to simultaneous improvement in the other under certain conditions. Similar results were also reported in lentil [14,15], whereas non-significant correlation was detected under E4, E5, and E6. The presence of phytic acid is a major constraint to iron and zinc intake. Our results in this regard showed non-significant correlation with both iron and zinc concentration under environments E1, E2, E4, E5 and E6, which supports our previous findings. Interestingly, under E3 growing conditions, phytic acid revealed a significant negative correlation with iron, suggesting that under extreme stress, iron bioavailability might be affected by changes in phytic acid metabolism. Both phytic acid/micronutrient ratios revealed a significant negative association with iron and zinc, while both ratios were positively and significantly correlated with phytic acid. These correlation patterns explain the reduction in iron and zinc bioavailability in lentil seed. These findings emphasize the need for breeding strategies that balance nutrient concentration with reduced anti-nutritional factors. In agreement with our findings, cooking time demonstrated a non-significant correlation with phytic acid under environments E2, E3, E4, E5, and E6. Supporting outcomes were published in lentil [18]. In contrast to our earlier findings, cooking time showed a significant negative correlation under environment E1 conditions.

The Principal Component Analysis (PCA) biplots provided key insights into the relationships among lentil genotypes and major agronomic and nutritional traits across normal, heat, and combined heat and drought stress conditions at Marchouch and Annoceur stations. At Marchouch station, the first two principal components explained 77.5% of the total variance. Under normal conditions, genotypes such as G3, G11, and G30 clustered with higher seed yield, hundred seed weight, iron, zinc, crude protein concentration, and longer cooking time. This suggests that optimal environments favor both productivity and nutritional quality, although higher protein levels may contribute to longer cooking times due to changes in seed structure affecting water absorption and softness.

Under heat stress, genotypes shifted centrally, indicating moderate declines in yield and nutrient accumulation but relative stability of protein concentration and cooking time. This resilience points to physiological mechanisms preserving protein synthesis and seed cooking quality even under stress. Similar trends have been reported by Sita et al. (2017) [19], who revealed that heat-tolerant lentil genotypes demonstrated stable reproductive development and maintained seed quality traits under heat stress due to efficient antioxidant defense systems, membrane stability, and chlorophyll retention [20]. Combined heat and drought stress caused genotypes such as G7 and G10 to associate with higher phytic acid levels and increased PA/Fe and PA/Zn ratios, reflecting a shift toward antinutritional storage compounds and a trade-off between stress adaptation and nutrient bioavailability [16].

At Annoceur station, a similar structure was observed, with PC1 and PC2 explaining 70.6% of the variance. Genotypes like G24, G20, and G34 under normal conditions showed strong associations with seed yield, hundred seed weight, protein, iron, and longer cooking time, confirming stable high performance across locations. Under combined heat and drought stress, genotypes again aligned with higher phytic acid-related traits, while heat stress alone was mainly linked to altered seed morphological features. Notably, although zinc concentration persisted under stress, its bioavailability was likely reduced due to elevated phytic acid levels.

Trait vector analysis across both sites showed strong positive correlations among antinutritional traits and strong negative correlations between phytic acid and mineral concentrations, indicating that abiotic stress not only limits yield but shifts seed metabolism toward less nutritionally favorable conditions, likely linked to oxidative stress responses [21]. These findings underscore the need for multi-trait selection strategies that combine grain yield, nutritional quality, cooking properties, and stress resilience. Genotypes like G3, G24, and G34, which balance these attributes, represent promising candidates for breeding climate-resilient, nutrient-dense lentil cultivars.

Selecting stable genotypes through assessment in diverse environments under specific stress conditions is crucial for varietal development. Genotypes environment interaction analysis was highly significant (*p* ≤ 0.001), implying that tested genotypes responded differently across environments. In this study, a significant portion of the variation was attributed to environmental factors, indicating that the environment used was different, which resulted in a significant variation in seed yield and nutritional quality traits. The mean yield varied significantly across environments, ranging from 5.15 g in E1 to 2.52 g E6, this variation reflected the difference in climatic conditions. The mean environment yield was positively related to both temperature and seasonal rainfall. E3 and E6 were the lowest yielding environments, whereas E1 and E4 were the highest yielding environments and had much seasonal rainfall. A large contribution of the environment was reported in lentil seed yield in earlier studies [17,18,19]. The variation in nutritional quality traits was also attributed to the effect of environmental conditions. In this study, environmental factors contributed to 98.95% of the total variance in protein content. Consequently, smaller proportions of the total variance were attributed to genotype (0.58%) and genotype × environment interaction (0.37%). However, genotype (G) effect was highly significant for protein content indicating contrastive performance among genotypes tested across environments. Several studies have also reported the impact of environment on lentil protein concentration [21,22,23]. Seed iron and zinc contents were also influenced by the environmental effect explaining 96.60% and 92.86% of the total variation, respectively. Our results are partially consistent with those previously reported by Darai et al. [15], who indicated that iron and zinc are highly sensitive to environmental fluctuations. Similar studies reported that both micronutrients exhibited considerable environmental interactions [13,24]. On the other hand, our results revealed that genotype (G) contributed less to the total variance for iron and zinc contents. Our results are consistent with previous finding, where genotype (G) explained the least of the total variance for micronutrients including iron and zinc [14,16]. Long cooking time is one of major limitations to the consumption and utilization of legumes in Africa [25,26]. Storage conditions, particularly high temperature and high relative humidity were previously reported to cause the hard-to-cook (HTC) defect. Our results revealed that cooking time in lentil was widely influenced by environmental effect, accounting for 95.36%. Similarly, environment explained 96.71% of the total variation in the phytic acid in lentil. Thavarajah et al. [27] reported that rising temperatures induced a significant increase in phytic acid in lentil seeds, which is consistent with our result. In addition, a highly significant increase in phytic acid was also observed in response to water scarcity regime in lentil [28]. In our study, G × E interaction explained the least of total variation for all tested quality traits suggesting the potential to develop stables genotypes with a combination of desirable nutritional quality traits. Furthermore, little variation in the genotype was observed due to factors such as variations in rainfall and temperature, resulting in distinct patterns of genotypic performance.

In this study, we identified the optimum genotype using the mean versus stability view of the GGE biplot. The mean performance and stability of 36 genotypes was assessed through the two PCAs and the projections from the abscissa toward the average environment coordinate ordinates (AEC). The abscissa and ordinate of the Average Environment Coordinate (AEC) are two lines that pass through the origin of the biplot, based on singular value partitioning (SVP = 1). The AEC abscissa is represented by a single-direction arrow, indicating the ideal genotype’s main effect, while the AEC ordinate, with a single arrow extending outside the biplot, highlights the greater mean performance of each genotype (Figure 4). The projection magnitude into the AEC ordinate determines the stability of the genotypes; consequently, a greater projection regardless of the direction signifies greater instability in the genotype.

For seed yield, iron, zinc, phytic acid, crude protein, and cooking time the shortest projection was identified for genotypes G26, G3, G19, G2, G3, and G12, respectively, and consequently identified as the most stable. Genotypes G9, G11, G6, G24, G21, and G32 had the longest projection and were, therefore, recognized as the most unstable. According to Alam et al. [29], an ideal genotype is characterized by both a high mean for the tested trait and significant environmental stability. Our findings revealed that E1 was the most desirable environment for seed yield and cooking time, while E4 and E2 were regarded as the ideal environment for iron and zinc content. Similarly, E5 was the most desirable environment for phytic acid and crude protein. The “which-won-where biplot distinguishes variations in mega-environments, indicating environments suitable for genotype adaptability, the top-performing genotypes within each mega-environment, and the ideal genotype exhibiting both high agronomic performance and stability [30]. In this study, different mega-environments were identified for each trait, and each mega-environment has different winning genotypes. This helps in understanding the complex genotype–environment interaction within a specific region. Additionally, genotypes specifically adapted to a particular mega-environment make it easier for breeders to develop environment-specific genotypes [31,32]. As stability analysis for yield becomes integral to plant breeding programs, the development of new and advanced models aims to enhance our understanding of genotype-environment interactions. Interestingly, the results from GGE analysis were largely consistent in identifying stable genotypes for each mega-environment, which aligns with previous studies using the GGE biplot to estimate G × E interaction of lentil genotypes [33,34]. Our study provides detailed information on the impact of heat and combined heat and drought stresses on seed yield and nutritional quality traits of lentil genotypes. The observed genotype × environment interaction indicates the need for further multi-year and multi-location trials to validate the stability and adaptability of selected genotypes. In addition, combining genomic techniques such as genome-wide association studies (GWAS) and marker-assisted selection could help clarify the genetic basis of stress tolerance and nutritional quality traits. Together, these approaches could support the development of climate-resilient and nutrient dense lentil cultivars.

## 4. Materials and Methods

### 4.1. Plant Material

**Figure 7:**
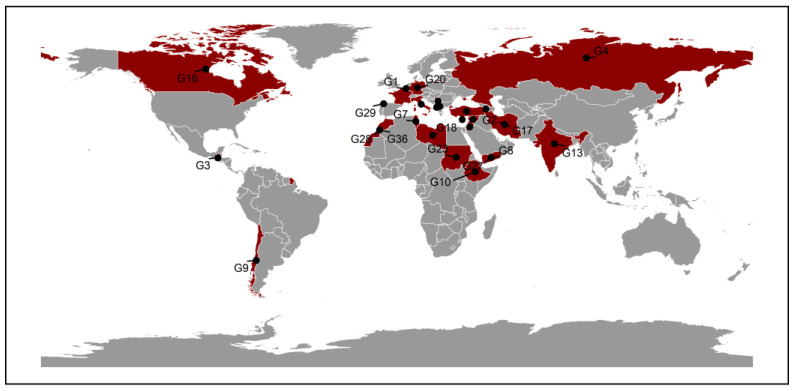
Geographic Origin of 36 Lentil Genotypes.

A total of 36 lentil genotypes were used in this study (Table S1), previously tested for nutritional quality traits and yield components [8,10]. Seeds were obtained from the genebank of the International Center for Agricultural Research in the Dry Areas, based in Rabat, Morocco (Figure 7).

### 4.2. Field Experiment

Experiment was conducted during 2019–2020 cropping season under two diverse environments in Morocco, corresponding to the following research station: ICARDA experimental research station at Merchouch and INRA research station at Announcer. The bioclimatic variation at the two locations offers differences in soil type, precipitation, and maximum and minimum temperature. Merchouch station is located 70 km south-east of Rabat (33.36° N 6.43° W, 390 m altitude). This station is situated in a semi-arid environment, with low rainfall and moderate temperatures during the winter and spring seasons. Daily weather data was recorded at the site. The annual average temperature was 16 °C, while the annual average rainfall was 323 mm. The soil at Merchouch is Vertisol and deficient in nitrogen with a pH level of 8.50.

Annoceur station is drought and cold station, winter in this station is characterized by cold and wet climate. The annual average temperature was 13 °C and ranged from −8 °C to 22 °C. The average annual precipitation was 337.4 mm, and the soils is gravelly calcareous clay with a pH of 8.35 (Table 4).

At both locations, experiments were conducted in a randomized complete block design (RCBD) with two replications. Each genotype was planted in a two-row plot, 1 m in length, with 30 cm spacing between rows. In each row, 25 seeds were manually sown at a depth of 2 cm, with 10 cm spacing between plants. In each site, three separate experiments involving the same set of genotypes were planted side by side on two sowing dates. Normal sowing was carried out during the last week of December 2019, while late sowing took place in the first week of February 2020 at all the experimental locations to impose heat stress during the flowering stage by exposing the crop to naturally higher temperature. For the late sowing, two different experiments were conducted, namely late planting with irrigation at field capacity throughout the crop period and late planting without irrigation during the reproductive phase allowing soil moisture to fall below 30% of field capacity, thereby imposing combined heat and drought stress. All recommended agronomic practices were followed to ensure a successful crop, including nutrient and weed management throughout the growing season (Table 5). Harvest at the Merchouch station occurred by the end of June, while at Annoceur station, harvest was in mid-July (Table 5).

### 4.3. Mineral Concentration

Five hundred milligrams from each sample was decomposed in a digestion block (QBlock series, Ontario, ON, Canada) using 6 mL concentrated (70%) nitric acid (HNO_3_). The mixture was heated for 60 min at 90 °C. A total of 3 mL of 30% hydrogen peroxide (H_2_O_2_) were added, and samples were further heated for 15 min at 90 °C until a colorless liquid was obtained. Subsequently, 3 mL of 6 M hydrochloric acid (HCl) was added. After cooling to room temperature, the volume was filtered and diluted to 1:10 using deionized water. Fe and Zn concentrations were determined using inductively coupled plasma-optical emission spectroscopy (ICP-OES) with an ICAP-7000 Duo (Thermo Fisher Scientific, Saint-Genis-Laval, ARA, France). Calibration curves for Fe and Zn were prepared by serial dilution in the range of 0.1 to 10 mg L^−1^. The total concentrations of Fe and Zn were validated by comparing the measurements to the NIST standard reference material.

### 4.4. Protein Concentration

Total nitrogen in seeds was determined using the micro-Kjeldahl method, following the procedure suggested by Baethgen et al. [35]. Three hundred grams of lentil flour was digested with a mixture of concentrated sulfuric acid (H_2_SO_4_), salicylic acid, and selenium (catalyst) at 300 °C for five hours. Seed protein concentration was determined by multiplying the nitrogen values by a factor of 6.25. Triple analyses were carried out on each sample.

### 4.5. Phytic Acid Concentration

The concentration of phytic acid in lentil seeds was determined using a Megazyme kit following the provided protocol [36]. One gram of ground lentil seed was digested with 20 mL of 0.66 M HCl solution in 50 mL falcon tubes and placed on a shaker for 15 h at room temperature. Afterward, 1 mL of the resulting extract underwent enzymatic reactions to release inorganic phosphorus (Pi) from phytic acid. The Pi was then reacted with ammonium molybdate to form 12-molybdophosphoric acid, which is reduced under acidic conditions to produce molybdenum blue. The amount of molybdenum blue formed correlates with the amount of Pi in the original sample and, thus, the phytic acid content. The absorbance of the molybdenum blue was measured at 655 nm using a UV-Visible spectrophotometer (T80 series, PG Instruments, Lutterworth, ENG, United Kingdom). Phosphorus solutions were prepared according to the Megazyme manual, using a standard phosphorus solution (24 mL, 50 µg/mL), and were treated as samples for the colorimetric phosphorus determination.

The amount of inorganic phosphorus (Pi) was calculated from the generated calibration curve, and the phytic acid concentration was estimated assuming that the measured phosphorus was exclusively released from phytic acid and accounted for 28.2% of phytic acid. This 28.2% factor corresponds to the precise stoichiometric proportion of phosphorus within the phytic acid molecule and enables the conversion of the measured phosphorus weight into an estimated phytic acid weight.

(1) $$\mathrm{Phytic}\;\mathrm{acid}=\frac{\mathrm{Phosphorus}\;[g/100\;g]}{0.282}$$

### 4.6. Phytic Acid/Iron and Phytic Acid/Zinc Molar Ratios

The molar ratios of phytic acid to iron and phytic acid to zinc were calculated using the following approach: the phytic acid content (PA) was divided by its molecular weight (660.04 g/mol), and the respective micronutrient (iron or zinc) content (MN) was divided by its molecular weight (65.4 g/mol for zinc and 55.85 g/mol for iron). The resulting ratios represent the relationship between phytic acid and each micronutrient.

### 4.7. Seed Size and Seed Shape Parameters

The hundred seed weight and seed geometry were evaluated through image analysis using the OptoAgri2, a high-speed seed counting device developed by Optomachine(Optomachine, Lyon, ARA, France). The OptoAgri2 system incorporates a high-resolution camera, selected based on grain size, a laboratory balance, and specialized software with an algorithm developed by OPTOmachine for image processing. This software enables the measurement of various seed attributes, such as area, perimeter, length, width, circularity, diameter, thickness, and rugosity. Additionally, it calculates the hundred seed weight.

### 4.8. Cooking Time

An automated Mattson Cooker (Mattson, Grand Rapids, MI, USA) was used to determine the cooking time for lentil seeds. This device featured a cooking rack with 25 holes, each accommodating a 2 mm tip diameter weighted plunger weighing 80 g. Before the cooking process, 2 g of lentil seeds were soaked in 50 mL of distilled water for 12 h at room temperature. Following the soaking period, 25 soaked lentil seeds were randomly distributed, with one placed in each of the 25 perforations on the rack. The tips of the plungers were positioned atop the seeds. The loaded Mattson Cooker was then immersed in a 2 L beaker filled with 1.5 L of boiling distilled water, and the beaker was covered to minimize evaporation. The entire setup was heated directly using a hot plate at 390 °C, and the cooking time was tracked using the Easy CT program.

Cooking time represented the duration required for 80% of the lentil seeds to be pierced, indicating they were adequately cooked. As the plungers penetrated the seeds and encountered the sensor on the fourth rack, the program automatically recorded the cooking time.

### 4.9. Statistical Analyses

Statistical analyses and data visualization were performed utilizing R version 4.2.1. Summary data was reported as range and mean values ± standard deviation. Boxplots of seed yield and nutritional quality traits were visualized using the ‘dplyr’, ‘tidyverse’ and ‘ggplot2’ packages. A two way analysis of variance (ANOVA) was used to assess the effects of genotypes (G), environment (E) and genotype x environment interaction (GEI) on the measured traits. Prior to ANOVA, assumptions of normality and homogeneity of variance were evaluated using the Shapiro–Wilk test and Levene’s test, respectively.

The mean significance of seed yield and nutritional quality traits was determined using Tukey’s test for mean comparison (*p* ≤ 0.05) using the ‘agricolae’ package. Network correlations between seed yield and nutritional quality traits were analyzed through Pearson’s correlation coefficient (*p* ≤ 0.05) using the ‘GGally’ package.

The agricolae package in R was used to perform Genotype-by-Environment Interaction (GGE) analysis. GGE biplot analysis is a method that helps in evaluating the interaction between genotypes and environments, identifying stable genotypes across different environments, and selecting the best-performing genotypes under specific environmental conditions. This analysis helps in understanding the adaptability and stability of genotypes and is useful for plant breeders when selecting suitable varieties for different environmental conditions.

## 5. Conclusions

The current study successfully provided valuable insights into the genetic variability and stability of 36 lentil genotypes for seed yield, nutritional quality (iron, zinc, protein), anti-nutritional factors (phytic acid), and cooking time under normal, heat, and combined heat-drought stress conditions. Environmental factors had a dominant effect on trait expression, especially under stress, with notable reductions in yield, protein, micronutrient content, and cooking time in E3 and E6. In contrast, favorable environments like E1 and E4 supported higher yield and better nutritional quality. Key trait correlations were identified, with hundred seed weight positively associated with seed yield, and iron and zinc levels often negatively correlated with phytic acid and its molar ratios highlighting the need to balance nutritional enhancement with anti-nutritional reduction in breeding programs.

Genotype-by-environment interaction (GEI) analysis confirmed differential genotype responses, reflecting the challenge of identifying stable, broadly adapted genotypes. Traits like phytic acid and cooking time were highly sensitive to environmental stress, yet genotypes such as G3, G26, G32, and G36 stood out for their stability and strong performance across environments, making them promising candidates for biofortification and climate-resilient breeding. GGE biplot analysis helped identify ideal genotypes and mega-environments, with the “which-won-where” pattern revealing both general and specific adaptations. Environments like E1, E4, and E5 were particularly informative for selection.

To meet breeding targets for lentil improvement under climate stress, these top-performing genotypes should be prioritized in further multi-year field trials. Evaluating their agronomic and nutritional traits using the integrated approaches applied in this study, supported by the application of advanced genomic tools, will strengthen efforts to develop lentil varieties that combine yield stability with high nutritional quality and stress resilience.

## Figures and Tables

**Table 1:** Analysis of variance (ANOVA) for seed yield and seed shape parameters of 36 Lentil genotypes tested at two locations under three treatments.

Source of Variation	df	SY	HSW	SL	SW	SA	SP	SD
Marchouch								
G	35	3.06 **	2.03 **	0.70 **	0.84 **	32.81 **	10.49 **	0.65 **
T	2	99.57 **	56.47 **	0.17 **	0.37 **	2.67 **	0.76 **	0.17 **
G × T	70	0.83 **	0.65 **	0.63 **	0.65 **	34.46 **	10.59 **	0.62 **
Residual	107	1.91 × 10^−3^	9.05 × 10^−4^	1.95 × 10^−3^	1.93 × 10^−3^	3.20 × 10^−2^	3.17 × 10^−2^	2.13 × 10^−3^
Annoceur								
G	35	1.33 **	0.89 **	0.26 ns	0.23 ns	16.00 ns	10.85 **	0.30 ns
T	2	34.64 **	11.21 **	0.03 ns	1.02 *	12.28 ns	32.35 **	0.16 ns
G × T	70	1.18 **	0.76 **	0.33 ns	0.32 *	19.56 *	11.80 **	0.38 *
Residual	107	0.23	0.19	0.25	0.20	10.67	3.22	0.21
Combined								
G	35	2.09 **	1.21 **	0.42 **	0.46 **	21.12 **	9.73 **	0.44 **
L	1	44.33 **	33.48 **	2.86 **	0.27 ns	109.29 **	93.75 **	3.14 **
T	2	128.66 **	60.89 **	0.05 ns	0.09 ns	19.69 **	27.1 ns	0.46 ns
G × L	35	2.25 **	1.70 **	0.50 **	0.60 **	26.27 **	11.15 **	0.49 **
G × T	70	1.10 **	0.52 *	0.53 **	0.54 **	28.29 **	11.07 **	0.53 **
Residual	272	0.36	0.35	0.19	0.19	10.31	4.19	0.19

SY, Seed yield; HSW, hundred seed weight; SL, Seed length; SW, Seed width; SA, Seed area; SP, Seed perimeter; SD, Seed diameter; G, Genotype; L, Location; T, Treatment; G × L, Genotype location interaction; G × T, Genotype treatment interaction. * and ** at 0.05 and 0.001 probability levels. df, degrees of freedom. ns, non-significant.

**Table 2:** Analysis of variance (ANOVA) for seed nutritional quality traits of 36 Lentil genotypes tested at two locations under three treatments.

Source of Variation	df	Fe	PA/Fe	Zn	PA	PA/Zn	CP	CT
Marchouch								
G	35	3.01 **	13.50 **	0.54 **	0.03 **	14.15 **	2.08 **	11.47 **
T	2	82.31 **	1066.61 **	8.09 **	2.65 **	1512.29 **	290.32 **	416.32 **
G × T	70	0.91 **	3.66 **	0.37 **	0.01 **	7.67 **	1.28 **	3.73 **
Residual	107	6.10 × 10^−3^	1.80 × 10^−2^	3.34 × 10^−3^	1.13 × 10^−4^	4.03 × 10^−2^	9.45 × 10^−2^	6.93 × 10^−3^
Annoceur								
G	35	2.71 **	9.70 **	0.18 ns	0.03 **	13.64 **	4.99 **	7.55 **
T	2	187.61 **	770.12 **	2.43 **	0.78 **	570.28 **	787.32 **	424.1 **
G × T	70	0.65 **	2.89 **	0.29 *	0.01 **	7.23 *	2.16 *	3.81 **
Residual	107	0.20	1.02	0.17	0.00	4.90	1.44	1.33
Combined								
G	35	3.41 **	14.27 **	0.36 **	0.03 **	16.38 **	3.68 **	12.59 **
L	1	65.02 **	44.08 **	28.45 **	0.09 **	580.86 **	682.88 **	33.68 **
T	2	255.83 **	1839.26 **	9.40 **	3.24 **	1991.62 **	1021.85 **	860.99 **
G × L	35	2.62 **	9.84 **	0.37 **	0.03 **	12.07 **	3.78 **	6.17 **
G × T	70	0.96 **	4.14 **	0.31 **	0.02 **	8.68 **	1.65 ns	4.44 **
Residual	272	0.34	1.02	0.15	0.01	3.95	1.67	1.31

Fe, Iron; Zn, Zinc; PA, Phytic acid; PA/Zn, Phytic acid/Zinc ratio; PA/Fe, Phytic acid/Iron ratio; CT, Cooking time; CP, Protein concentration; G, Genotype; L, Location; T, Treatment; G × L, Genotype location interaction; G × T, Genotype treatment interaction. * and ** indicate significance at 0.05 and 0.001 probability levels. df, degrees of freedom. ns, non-significant.

**Table 3:** Range and mean performance for seed yield, seed size, and shape parameters of thirty-six lentil genotypes tested across six environments.

E	Descreptive	SY	HSW	SL	SW	SA	SP	SD
E1	Range	1.04–6.87	0.69–4.9	0.61–5.83	0.61–5.41	4.36–24.39	2.46–21.91	0.61–5.47
	Mean ± SD	5.13 ^a^ ± 2.97	3.50 ^a^ ± 1.8	4.70 ^a^ ± 3.57	4.43 ^a^ ± 3.27	16.16 ^a^ ± 8.85	17.59 ^a^ ± 12.98	4.48 ^a^ ± 3.35
E2	Range	0.82–5.66	0.79–4.11	0.52–6.05	0.56–5.63	3.79–25.27	2.11–22.76	0.50–5.70
	Mean ± SD	3.91 ^b^ ± 2.42	2.55 ^b^ ± 1.1	4.6 ^b^ ± 3.81	4.29 ^b^ ± 3.19	15.81 ^b^ ± 10.43	17.39 ^b^ ± 14.04	4.39 ^b^ ± 3.64
E3	Range	1.02–4.58	0.89–3.2	3.73–5.92	2.99–5.56	9.98–25.39	13.77–22.26	3.5–5.59
	Mean ± SD	2.78 ^c^ ± 0.77	1.73 ^c^ ± 0.74	4.65 ^c^ ± 0.58	4.35 ^c^ ± 0.62	15.86 ^b^ ± 4.18	17.46 ^c^ ± 2.31	4.41 ^c^ ± 0.57
E4	Range	2.36–5.86	1.07–3.70	3.96–5.71	3.38–5.25	10.88–24.57	14.55–27.54	3.40–5.58
	Mean ± SD	4.04 ^a^ ± 0.95	2.46 ^a^ ± 0.72	4.78 ^a^ ± 0.46	4.25 ^b^ ± 0.47	17.45 ^a^ ± 3.97	19.27 ^a^ ± 3.77	4.65 ^a^ ± 0.56
E5	Range	1.76–4.73	1.05–3.74	4.35–5.57	4.02–5.20	13.30–22.13	16.11–20.93	4.10–5.30
	Mean ± SD	3.22 ^b^ ± 0.69	1.93 ^b^ ± 0.66	4.83 ^a^ ± 0.34	4.48 ^a^ ± 0.32	16.70 ^b^ ± 2.41	17.99 ^b^ ± 1.31	4.57 ^a^ ± 0.32
E6	Range	1.05–4.45	0.40–2.51	4.02–5.78	3.73–5.39	11.41–23.89	14.88–21.72	3.80–5.49
	Mean ± SD	2.52 ^c^ ± 0.82	1.60 ^c^ ± 0.63	4.81 ^a^ ± 0.44	4.47 ^a^ ± 0.42	16.62 ^b^ ± 3.10	17.95 ^b^ ± 1.70	4.56 ^a^ ± 0.42

SY, Seed yield; HSW, hundred seed weight; SL, Seed length; SW, Seed width; SA, Seed area; SP, Seed perimeter; SD, Seed diameter; E, Environment; SD, Standard deviation; E, Environment. Different letters (a–c) indicate significant differences at *p* < 0.05 (Tukey’s test).

**Table 4:** Agro-climatic and soil characteristics of experimental sites during 2019–2020 cropping season.

Parameter	Merchouch (ICARDA)	Annoceur (INRA)
Coordinates	33.36° N, 6.43° W	33.68° N, 4.85° W
Altitude	390 m	1350 m
Climate	Semi-arid	Cold and wet
Annual Average Temperature	16 °C	13 °C
Annual Average Rainfall	323 mm	337.4 mm
Soil Type	Vertisol, nitrogen-deficient	Gravelly, calcareous clay
Soil pH	8.50	8.35

ICARDA: International center of agriculture research in dry areas, INRA: National institute of agricultural research.

**Table 5:** Description of experimental treatments at each research station.

Code	Location	Description	Irrigation Timing
E1	Merchouch	Normal planting	Regular irrigation as per crop needs
E2	Merchouch	Late planting + irrigation	Irrigation maintained during reproductive stage (Field capacity)
E3	Merchouch	Late planting + no irrigation	No irrigation during reproductive stage (Drought stress)
E4	Annoceur	Normal planting	Regular irrigation as per crop needs
E5	Annoceur	Late planting + irrigation	Irrigation maintained regularly during reproductive stage (Field capacity)
E6	Annoceur	Late planting + no irrigation	No irrigation during reproductive stage (Drought stress)

E: Environment.

## Data Availability

The data are available within the article and the Supplementary Materials.

## Supplementary Materials

The following supporting information can be downloaded at: https://www.mdpi.com/article/10.3390/plants14132019/s1, Table S1: List of 36 tested genotypes, their source and country of origin; Table S2: Range and mean performance for seed nutritional quality traits and micronutrient bioavailability of thirty-six lentil genotypes tested across six environments; Table S3. Correlation coefficients among different trait combinations based on 36 lentil genotypes evaluated under normal, heat stress, and combined heat and drought stress at Marchouch (above the diagonal) and Annoceur (below the diagonal) research stations; Table S4. Summary of high-performing and stable lentil genotypes across tested environments (E1–E6).
