# Supplementary material for: Variation of Seed Yield and Nutritional Quality Traits of Lentil (Lens culinaris Medikus) Under Heat and Combined Heat and Drought Stresses
Source: Plants (Basel). 2025 Jul 1;14(13):2019. doi: 10.3390/plants14132019 (PMC12251599; doi:10.3390/plants14132019)
Supplement: Supplementary file 1 [file plants-14-02019-s001.zip › plants-3667967-supplementary.pdf]

Table S1: List of 36 tested genotypes, their source and country of origin.

IG	Origin	ID	Pedigree	DOI	Latitude	Longitude
**ILL 224**	BEL	PI 238758	NA	10.18730/5N97A	NA	NA
**ILL 257**	IRN	PI 289069	NA	10.18730/5NA64	36.8	54.4
**ILL 494**	GTM	PI 311107	NA	10.18730/5NHAA	14.6	-90.7
**ILL 597**	RUS	PI 343025	NA	10.18730/5NM1Q	54.3	48.3
**ILL 619**	ARM	PI 345638	NA	10.18730/5NMG1	40.8	43.8
**ILL 624**	MKD	PI 357226	NA	10.18730/5NMK4	41.4	21.9
**ILL 918**	TUN	NEL 918	NA	10.18730/5NX0E	36.8	10.2
**ILL 950**	YEM	PI 244046	NA	10.18730/5NY09	13.6	44
**ILL 956**	CHL	33-032-10127	NA	10.18730/5NY6F	-36.6	-72.1
**ILL 1959**	ETH	EL 102	NA	10.18730/5PWT~	9.1	38.7
**ILL 2181**	TUR	NA	ILL 182 selection	10.18730/5Q1AX	41.3	26.7
**ILL 2230**	YUG	NA	ILL 624 selection	10.18730/5Q2V4	NA	NA
**ILL 3517**	IND	LG 46	NA	10.18730/5RA54	26	85.9
**ILL 4345**	NA	5091	NA	10.18730/5S2ZN	NA	NA
**ILL 4471**	SYR	SAMPLE NO. 27	NA	10.18730/5S6S*	35.6	36.7
**ILL 4738**	CAN	ESTON	NA	10.18730/5SF10	NA	NA
**ILL 4791**	IRN	II-3-81	NA	10.18730/5SGPG	38.4	47.1
**ILL 4804**	LBY	II-3-135	NA	10.18730/5SH3X	33.5	35.4
**ILL 4841**	ALB	LENS 2	NA	10.18730/5SJ8X	40.7	20.6
**ILL 4881**	DEU	LENS 137	NA	10.18730/5SKG*	52.7	12.3
**ILL 5261**	JOR	UJL 42	80SH S19 UJL 32	10.18730/5SYGE	32.4	35.9
**ILL 5416**	ITA	NA	NA	10.18730/5T3BN	41.3	15.2
**ILL 5505**	SDN	NA	NA	10.18730/5T61~	19.2	30.5
**ILL 5509**	SYR	74TA 22	ILL 31 selection	10.18730/5T650	36.2	37.2

**ILL 5595**	SYR	78S 26030	ILL 25 selection	10.18730/5T8VC	33.5	36.3
**ILL 5968**	CYP	NA	NA	10.18730/7M0YS	34.7333	32.7333
**ILL 6281**	YEM	NA	NA	10.18730/7Q6CF	NA	NA
**ILL 6493**	MAR	NA	NA	10.18730/7QD5A	32.0833	-8.53333
**ILL 6528**	FRA	NA	NA	10.18730/7QE88	NA	NA
**ILL 6644**	SYR	NA	NA	10.18730/7QHTB	37.0292	41.5392
**ILL 6870**	SYR	NA	NA	10.18730/7SFMW	35.7667	36.65
**ILL 7084**	ITA	NA	NA	10.18730/7SNV5	37.5	15.0667
**Bichette**	JOR	NA	NA	10.18730/5T7TG	32.0667	36.1
**Chakkouf**	TUR	NA	NA	10.18730/7MEMK	37.1667	38.7833
**LSI88**	NA	NA	NA	NA	NA	NA
**Zaaria**	MAR	NA	NA	10.18730/8VS93	NA	NA

IG, ICARDA Germplasm; ID, Identifier; DOI, Digital object identifier; NA, Not available.

BEL, Belgium; IRN, Iran; GTM, Guatemala; RUS, Russian Federation; ARM, Armenia; MKD, Macedonia; TUN, Tunisia; YEM, Yemen; CHL, Chile; ETH, Ethiopia; TUR, Turkey; YUG, Yugoslavia; IND, Indian; SYR, Syria; MAR, Morocco; CAN, Canada; LBY, Libya; ALB, Albania; DEU, Germany; JOR, Jordan; ITA, Italy; SDN, Sudan; CYP, Cyprus; FRA, France.

Table S2. Range and mean performance for seeds nutritional quality of thirty-six lentil genotypes tested across six environments.

E	Descriptive	Fe	Zn	PA	PA/Fe	PA/Zn	PC	CT
E1	Range	1.09-11.02	0.48-6.81	0.09-0.99	1.35-11.3	1.86-17.22	0.87-31.8	2.37-15.03
	Mean ± SD	8.7^{a}±7.21	5.89^{a}±5.04	0.82^{a}±0.54	8.05^{a}±5.09	13.84^{a}±8.93	29.68^{a}±28.52	9.82^{a}±6.13
E2	Range	0.85-9.64	0.43-6.74	0.08-1.20	1.53-13.87	1.70-20.55	0.83-29.43	1.22-9.87
	Mean ± SD	7.63^{b}±6.08	5.66^{b}±4.77	0.98^{b}±0.81	11.10^{b}±8.16	17.29^{b}±13.86	27.36^{b}±25.2	7.04^{b}±4.85
E3	Range	5.38-8.11	4.47-6.67	0.97-1.41	10.46-20.48	16.2-27.02	24.01-27.42	2.00-9.13
	Mean ± SD	6.56^{c}±0.71	5.23+±0.47	1.20^{c}±0.11	15.69^{c}±2.50	22.92^{c}±2.90	25.68^{c}±0.93	5.03^{c}±1.53
E4	Range	7.14-11.86	4.29-6.37	0.62-1.10	12.21-5.17	11.66-22.69	27.13-31.24	5.30-14.21
	Mean ± SD	10.22^{a}±1.06	5.24^{a}±0.46	0.93^{a}±012	7.80^{a}±1.53	17.67^{a}±2.43	28.96^{a}±1.15	9.68^{a}±2.60
E5	Range	6.07-9.91	4.26-6.10	0.82-1.44	7.40-15.00	15.78-25.05	22.12-28.31	4.15-8.71

	Mean ± SD	8.46^{b}±0.80	5.11^{a}±0.35	1.10^{b}±0.17	10.36^{b}±0.26	19.97^{b}±0.39	24.54^{b}±1.51	6.05^{b}±1.05
E6	Range	5.18-8.66	4.29-5.45	0.92-1.33	9.79-20.16	17.66-28.33	19.61-23.71	2.54-6.21
	Mean ± SD	6.71^{c}±0.77	4.85^{b}±0.32	1.15^{c}±0.09	14.74^{c}±035	23.67^{c}±0.40	21.74^{c}±1.37	4.43^{b}±0.86

SY, Seed yield; Fe, Iron; Zinc; PA, Phatic acid; PA/Zn, Phytic acid/Zinc ratio; PA/Fe, Phytic acid/Iron ratio; PC, Protein concentration; CT, cooking time; SD, Standard deviation. E, Environment; SD, Standard deviation.

Table S3. Correlation coefficients among different trait combinations based on 36 lentil genotypes evaluated under normal, heat stress, and combined heat and drought stress at Marchouch (above the diagonal) and Annoceur (below the diagonal) research stations.

Traits	SY	HSW	Fe	Zn	PA	PC	CT
				NR			
SY	1	0.68**	4.60E-03	0.06	0.02	-0.28	-0.04
HSW	0.84**	1	-4.50E-03	-0.06	0.06	-0.14	0.13
Fe	0.50**	0.41*	1	0.46**	-0.08	2.48E-03	-0.21
Zn	0.18	0.19	0.21	1	-0.02	-0.06	-0.05
PA	-0.16	-0.18	-0.09	0.07	1	0.13	-0.35*
PC	-0.07	-0.13	-0.16	0.21	0.07	1	0.29
CT	0.09	0.05	0.04	0.06	0.05	-0.06	1
				HT			
SY	1	0.68**	-0.27	-0.16	-0.01	0.11	-0.20
HSW	0.73**	1	-0.39*	-0.16	0.11	0.14	-0.17
Fe	0.14	0.10	1	0.48**	-0.01	-0.03	-0.08
Zn	-0.07	-0.04	0.42*	1	0.11	0.20	-0.03
PA	-0.07	-0.19	-0.13	0.08	1	0.25	0.15
PC	-0.03	-0.05	-0.17	0.04	-0.15	1	0.17
CT	-0.03	-0.12	0.35*	0.01	-0.15	0.10	1
				HT+HD			
SY	1	0.80**	-0.29	-0.04	0.05	-0.04	0.08
HSW	0.84**	1	-0.41*	-0.10	0.18	-0.05	0.01
Fe	0.50**	0.41*	1	0.41*	-0.32	-0.32	-0.09

Zn	0.18	0.19	0.21	1	-0.08	0.01	0.25
PA	-0.16	-0.18	-0.09	0.07	1	0.19	0.05
PC	-0.07	-0.13	-0.16	0.21	0.07	1	-0.10
CT	0.09	0.05	0.04	0.06	0.05	-0.06	1

SY, Seed yield; Fe, Iron; Zinc; PA, Phatic acid; PC, Protein concentration; CT, Cooking time; SD, Standard deviation; NR, Normal condition; HT, Heat stress; HT+DH, Combined heat and drought stresses.

Table S4. Summary of high-performing and stable lentil genotypes across tested environments (E1-E6)

Trait	High-Performing Genotypes	Environment(s)
SY	G34, G36, G27, G10, G4, G16, G2	E1, E2
	G9, G36, G29	E3, E6
	G17, G24, G21, G14, G20, G22, G12	E4, E5
Fe	G14, G34, G11, G29, G32, G3, G15, G6	E4–E6
	G6, G20, G33, G1, G19, G22, G3, G4, G12	E1–E3
Zn	G32, G10, G36, G3, G4, G13	E1, E5, E6
	G16, G11, G14, G33, G5, G9, G34, G12	E2, E3
	G23, G8, G7, G6, G26, G30, G2, G25	E4
PA	G13, G34, G33, G2, G1, G10, G36, G30, G22, G21	E1, E5, E6
	G13, G1, G2, G9, G32, G11, G36, G22	E2, E3
	G34, G10, G15, G5, G33, G7, G8, G16	E4
PC	G21, G11, G25, G8, G29, G14, G33, G15	E1, E2, E4
	G15, G24, G31, G23, G22, G5, G28, G32, G13	E5, E6
	G7, G12, G26, G1, G16, G3, G9, G2	E3
CT	G25, G33, G9, G10, G23, G19, G27, G2	E4–E6

G28, G32, G2, G22, G27, G5	E1, E2, E4

SY, Seed yield; Fe, Iron; Zinc; PA, Phatic acid; PC, Protein concentration; CT, cooking time; SD, Standard deviation. E, Environment; G, Genotype.
